# Unwanted scenario of medical journals’ future: Artificial intelligence and medical writing

**DOI:** 10.5935/0004-2749.2025-0090

**Published:** 2025-04-25

**Authors:** Shigeki Matsubara

**Affiliations:** 1 Department of Obstetrics and Gynecology, Jichi Medical University, Tochigi, Japan; 2 Department of Obstetrics and Gynecology Koga Red Cross Hospital, 1150 Shimoyama, Koga, Ibaraki 306-0014, Japan; 3 Medical Examination Center, Ibaraki Western Medical Center, 555 Otsuka, Chikusei, Ibaraki 308-0813, Japan

Dear Editor,

Two recent articles on generative artificial intelligence (GenAI) in academic writing warrant attention. Liu et al.’s review^(1)^ stands out, and Ambrósio et al.’s^(2)^ insights are particularly astute. While both papers highlight GenAI’s impressive capabilities, they also express concerns about its drawbacks, particularly the risk of over-reliance on it. To complement their concern, l outline a hypothetical, undesirable future scenario that GenAI might bring to academic publishing. Rather than offering prescriptive solutions, my aim is to stimulate a broader conversation about the implications of GenAI in academic writing.

The current state of GenAI use in writing can be summarized as follows:

1. GenAI regulations in writing are not yet standardized^(1^, ^3)^. While many journals allow its use for “readability improvement”, the term is open to interpretation. Some authors might use ChatGPT to generate entire manuscripts from key findings, while others employ it solely as a grammar checker. This creates room for non-declaration of GenAI use.
2. Tools designed to detect AI-generated manuscripts are imperfect and likely to remain so^(4)^, especially when humans extensively edit AI-generated texts.
3. Given appropriate input, AI can generate manuscripts that are as readable as those written by humans^(5)^. Many authors, especially less experienced ones, are likely to rely on AI^(4^, ^5)^, and this tendency is expected to continue.
4. The push for work-life balance constrains the time physicians can dedicate to sentence-by-sentence reflection, making AI use in academic writing inevitable, especially for non-native authors.

These factors create a complex unresolved issue that stakeholders will continue to grapple with^(4)^. The challenge lies in striking a balance between genuine human writing and reliance on AI^(1^, ^4)^. Achieving a universally acceptable balance seems elusive, as opinions on the matter tend to be highly subjective. What will the future of paper writing look like? l will illustrate one possible, albeit extreme scenario that may, paradoxically, help clarify or even resolve this conundrum.

In the future, medical papers may resemble a “dictionary”, losing the element of “how it’s written”. Traditionally, we have valued both content (“what is written”) and tone (“how it is written”) in medical papers. An extreme example of the latter is seen in “human touch” (anecdotes, experiential insights, and proverbial wisdom). This touch enriches manuscripts^(5)^. However, we may need to abandon such touch or tone.

A dictionary? Nobody minds its “flat” descriptions. We do not expect a writing tone from it: accuracy and thus “what is written” matters. lf “dictionary-like papers” is difficult to imagine, consider an anatomy textbook, where most pages feature straightforward factual statements.

I anticipate that future papers will become more formulaic and dry, resembling a dictionary or an anatomy textbook. For instance, essential information may be condensed into bullet-point formats (Table 1), replacing the traditional IMRaD (Introduction, Methods, Results, and Discussion) style. Once adapted, this style may enable readers to rapidly grasp core points, much like referencing a dictionary.

**Table 1:** Hypothetical structure of “future” original articles

1. What is already known: One, two, three.
2. What is yet unknown: One, two, three.
3. What was clarified in this study (among point 2).
4. Methods and Results: Similar to IMRaD, but focusing only on key points; less important details go into supplementary materials.
5. Short description of strengths and weaknesses.
6. A brief summary of what is new. If no new findings, what has been reconfirmed?
7. A “take-home message” at the end.

IMRaD: Introduction, Methods, Results, and Discussion.**This is only an example.** In this structure, readers can start from any section based on what they want to know at that moment. Even today, when we urgently need to understand a paper’s content, we often scan it like a dictionary, searching for points **(1)–(7),** which are sometimes embedded within the IMRaD structure.

A bullet-point style manuscript may require minimal writing finesse, freeing authors from concerns about tone and style. By excluding these elements, reliance on AI decreases, leaving only the content. Writing bullet points is straightforward, making AI assistance less necessary. Thus, AI reliance will naturally wane. I am not dismissing the value of IMRaD; with over 590 PubMed-indexed papers under my belt, I respect its merits, forged through the persistent efforts of many. Still, I foresee a shift. IMRaD may not be eternal.

This bullet-point style comes with a cost: the loss of individual tone and writing flair. I fervently hope Editorials, Opinions, and Letters remain the domain of human writers. In these “thought-expressing pieces”, “how it is written” holds significant weight^(5)^. If original papers lose this element, such pieces will become even more important.

Perhaps journals will resemble a “column among data books”, with thought-expressing pieces serving as columns where the role of authors as “writers” is preserved.

I expect the academic community to find solutions that balance AI’s benefits with preserving individuality in writing, rendering my concerns moot. I hope this is the case. Even in a worst-case scenario, I believe that individuality can still be expressed in subtle details. Just as high-school students in identical uniforms can express themselves through small touches, like a red lining, humans are wired to notice and appreciate these nuances.
