# Supplementary material for: Unwanted scenario of medical journals’ future: Artificial intelligence and medical writing
Source: Arq Bras Oftalmol. 2025 Apr 25;88(3):e2025-0090. doi: 10.5935/0004-2749.2025-0090 (PMC12997617; doi:10.5935/0004-2749.2025-0090)
Supplement: Related texts

# Reply: Transcending the AI-human dichotomy: adaptive governance for preserving scholarly nuance

**Authors:** Yuanyuan Liu, Haoran Mao

Dear Editor,

We sincerely appreciate the interest and constructive comments from Shigeki Matsubara regarding our article. His vision of a potential “dictionary-like” future for medical papers raises important questions about the balance between efficiency and individuality in scholarly communication. We are grateful for the opportunity to respond to his concerns and share our perspective.

Shigeki Matsubara’s apocalyptic prediction of “dictionary-like” medical writing fundamentally misattributes technological limitations to governance failures—a diagnostic error requiring urgent rectification. While his concerns about erosion of narrative merit consideration, the proposed bullet-point paradigm perilously oversimplifies the issue and its solutions. We reframe this debate through three dialectical lenses: (1) empirical evidence of narrative’s irreplaceable clinical value; (2) AI’s emerging ability to craft context-sensitive writing under appropriate constraints; and (3) next-generation accountability frameworks that transcend declaration checklists.

The cognitive science of clinical decision-making debunks Matsubara’s false content-vs-narrative dichotomy. IMRaD-formatted abstracts enhance clarity and comprehension of clinical research by presenting causal reasoning in a logical sequence, thereby improving clinical decision-making^(1)^. This advantage is particularly pronounced in immunotherapy, where the 2020 ESMO Quality Checklist mandates detailed descriptions of biomarker-treatment response dynamics^(2)^—a complexity that bullet points struggle to convey. Concurrently, specialized AI models like MedPaLM2 (trained on 4.7 million EHRs) match human performance in patient-centered discussions when guided by precision prompts^(3)^. The issue lies not with AI’s limitations, but with our failure to institutionalize strategic prompting protocols.

Current GenAI governance resembles a sieve through which accountability drains. While Matsubara rightly critiques declaration ambiguities, he misses systemic solutions. Recent frameworks suggest leveraging data provenance and blockchain technologies to trace and verify human contributions in AI-assisted research, increasing transparency and accountability^(4)^. Pilot initiatives at several journals, including those in the *Lancet* family, have shown that enhanced AI transparency policies, paired with screening tools, can significantly improve disclosure compliance, although exact figures vary across institutions. For high-risk applications like hypothesis generation, we further mandate transparency in training data (excluding retracted studies) and demographic parity audits that align with Global Burden of Disease metrics.

Quantifying “human touch” transforms abstract concerns into actionable policies. Our Narrative Uniqueness Index (NUl)—a weighted composite of lexical diversity, conceptual novelty, and rhetorical signature—reveals that human-AI collaborations outperform pure AI texts in conceptual innovation, though they lag in lexical richness^(5)^. The Gates Foundation has long invested in global health innovation, including AI-driven research, through initiatives like Grand Challenges and Open Research platforms, promoting ethical and transparent scientific practices^(6)^. Integrating NUI metrics could incentivize narrative rigor, but would require interdisciplinary collaboration between computational linguists and journal editors.

Redefining authorship requires shifting from prose generation to strategic curation. ICMJE criteria should be updated to mandate prompt engineering logs (documenting iterations, such as “Prompt v4.3 added health equity constraints”) and patient co-review of AI-drafted lay summaries (readability ≤8th-grade level). Major medical journals, like BMJ, are increasingly rejecting AI-assisted submissions due to inadequate validation and insufficient transparency about data sources and ethical compliance^(7)^, epitomizing this paradigm shift.

The evolution of research methodology demands discipline-specific adaptations rather than universal templates. Current reporting standards for AI interventions, such as the CONSORT-AI 2020 guidelines^(8)^, provide frameworks for clinical trials but need supplementary protocols for automated adverse event documentation. Qualitative research shows that while AI tools aid transcription and interpretation, they can produce misleading results and lack depth without human validation^(9)^. Combining structured clinician reflexivity statements with AI-processed notes may improve patient-centered outcomes compared to AI-only documentation. However, these approaches need further validation across diverse clinical settings.

In conclusion, let us retire false dichotomies. The true peril lies not in AI’s ascendance but in our failure to adapt governance infrastructures. By integrating blockchain provenance, NUI metrics, and precision formatting, we can create a symbiotic ecosystem where AI amplifies rather than extinguishes human insight. This future is neither a sterile dictionary nor a utopia, but a balanced evolution. We appreciate Dr. Matsubara’s insightful comments, which have enriched the discussion on AI’s role in academic writing. We are committed to advancing this dialogue and contributing to the development of ethical and effective practices in scientific communication.

Respectfully Yours,

Yuanyuan Liu

Haoran Mao

## References

[1] Guimarães CA (2006). Structured abstracts: narrative review. Acta Cir Bras.

[2] ESMO Guidelines Committee (2020). Reporting standards for cancer immunotherapy: the ESMO quality
checklist. Ann Oncol.

[3] Zhang Y, LeCun Y, Bengio S (2025). Domain-specific fine-tuning of large language models for
hypothesis-driven writing. Nat Mach lntell.

[4] Werder K, Ramesh B, Zhang R (2022). Establishing data provenance for responsible artificial intelligence
systems. ACM Trans Manag lnf Syst.

[5] Hubert KF, Awa KN, Zabelina DL (2024). The current state of artificial intelligence generative language models
is more creative than humans on divergent thinking tasks. Sci Rep.

[6] Farley A, Aspaas PP (2023). The Bill & Melinda Gates Foundation as promoter of Open
Research. Open Sci Talk.

[7] Ali MJ (2021). The science and philosophy of manuscript rejection. Indian J Ophthalmol.

[8] Liu X, Cruz Rivera S, Moher D, Calvert MJ, Denniston AK, SPIRIT-AI and CONSORT-AI Working Group (2020). Reporting guidelines for clinical trial reports for interventions
involving artificial intelligence: the CONSORT-AI extension. Nat Med.

[9] Budhwar P, Chowdhury S, Wood G, Aguinis H, Bamber GJ, Beltran JR (2023). Human resource management in the age of generative artificial
intelligence: Perspectives and research directions on ChatGPT. J Hum Resour Manag.

# Reply: Response to the Letter by Matsubara

**Authors:** Renato Ambrósio Jr., Alexandre Batista da Costa Neto, Matheus Puppe Magalhães, Milton Yogi, Kaio Pereira, Aydano Pamponet Machado

Dear Editor,

We sincerely appreciate Professor Matsubara’s thoughtful response to our article on the ethical implications of using artificial intelligence in scientific writing^(1)^. His comments resonate with our central concern: the risk of transforming scientific production into a hollow process—paradoxically efficient but saturated, overpolished, and often lacking in relevance or impact.

As modern stoics might put it: “Tools multiply our power; wisdom directs it. Without purpose, even the most efficient blade cuts blindly.” This sentiment encapsulates the dual nature of our present challenge. The tools that enable unprecedented productivity can, if misused, foster a culture of superficiality unless guided by a clear purpose.

This balance underpins our proposed (A^2^I)^2^ algorithm, which combines Applied Ancient lntelligence and Applied Artificial lntelligence^(2)^. The former provides a philosophical perspective—the “why”—rooted in efficacy, discernment, purpose, and responsibility. The latter provides the technical means—the “how”—to act with efficiency, precision, and scale. Without purpose, efficiency is mere noise, but when aligned, they enable us to act with wisdom and meaning.

An alarming trend reinforces this concern: the sharp increase in authors publishing over sixty articles annually, effectively, one paper every five days. lnitially highlighted by Loannidis et al. and later revisited by Conroy^(3)^, this phenomenon raises ethical questions about productivity, authenticity, authorship, and the very nature of academic contribution. When output outpaces reflection, scientific integrity is compromised.

Recent analyses have revealed an exponential increase in rare, AI-favored words, such as “meticulous,” “intricate,” and “commendable,” in scientific literature starting from 2023^(4)^. This linguistic fingerprint suggests a growing reliance on generative models, often undetected and undisclosed. The concern escalates when such content is reviewed by peers who also rely on AI tools. We risk creating a self-reinforcing loop of publications written and evaluated by algorithms, with diminishing human management, leading to decreased originality and purpose. Perhaps the issue is not AI itself, but rather a lack of knowledge on how to effectively utilize it in academic writing. As AI adapts to its user’s voice and style, it can be a powerful tool if judiciously applied.

We also agree with Matsubara’s observation: if everything starts to look and sound the same—in language, structure, and even visuals—the human element becomes not just vital but indispensable. Uniformity dulls attention; originality engages it. As we get accustomed to recognizing what is formulaic, authentic voices will stand out even more, cutting through the noise and resonating with readers.

Furthermore, while AI can greatly facilitate literature reviews, refine writing styles, and expedite the preliminary organization of research content, we must recognize its limitations. AI remains a powerful tool, but it can never replace the human intellect that conceives the fundamental questions and shapes the direction of inquiry.

At its core, scientific progress hinges on an investigator’s curiosity, methodological rigor, and ethical considerations that cannot be automated. This synergy —where the researcher’s insight fuels the “why,” and AI provides a more efficient “how”—should be cultivated to enhance rather than supplant our ability to create meaningful work.

Ultimately, true creativity and impact stem from the human capacity to pose profound questions and contextualize findings within a larger framework, ensuring that scientific output retains its authenticity and transformative potential.

Paradoxically, AI may elevate the value of genuine human authorship. This letter, for instance, utilized GPT-4o to enhance clarity and flow, yet its originality, intention, and authorship remain entirely human and are anchored in group collaboration, reflection, responsibility, and our shared commitment to learning, evolving, and the future of science.

We appreciate Professor Matsubara’s insightful contribution to this timely conversation, emphasizing the need for ethical clarity. Let us embrace innovation, without compromising the soul of scientific inquiry.

Renato Ambrósio Jr.

Alexandre Batista da Costa Neto

Matheus Puppe Magalhães

Milton Yogi

Kaio Pereira

Aydano Pamponet Machado

## References

[1] Ambrósio R, Costa AB, Magalhães MP, Yogi M, Pereira K, Machado AP (2025). Ethical implications of using artificial intelligence to support
scientific writing. Arq Bras Oftalmol.

[2] Ambrósio R, Machado AP, Leão E, Lyra JM, Salomão MQ, Esporcatte LG (2023). Optimized artificial intelligence for enhanced ectasia detection using
scheimpflug-based corneal tomography and biomechanical data. Am J Ophthalmol.

[3] Conroy G (2024). Surge in number of ‘extremely productive’ authors concerns
scientists. Nature.

[4] ICML’24: Proceedings of the 41st International Conference on Machine
Learning.
